# Everywhere and for everyone: proportionate universalism as a framework for equitable access to community drug checking

**DOI:** 10.1186/s12954-022-00727-0

**Published:** 2022-12-20

**Authors:** Bruce Wallace, Thea van Roode, Piotr Burek, Dennis Hore, Bernadette Pauly

**Affiliations:** 1 Canadian Institute for Substance Use Research (CISUR), University of Victoria, P.O. Box 1700, STN CSC, Victoria, BC V8W 2Y2 Canada; 2 School of Social Work, University of Victoria, P.O. Box 1700, STN CSC, Victoria, BC V8W 2Y2 Canada; 3 Department of Chemistry, University of Victoria, P.O. Box 1700, STN CSC, Victoria, BC Canada; 4 Department of Computer Science, University of Victoria, P.O. Box 1700, STN CSC, Victoria, BC V8W 2Y2 Canada; 5 School of Nursing, University of Victoria, P.O. Box 1700, STN CSC, Victoria, BC V8W 2Y2 Canada

**Keywords:** Drug checking, Harm reduction, Substance use, Fentanyl, Overdose, Health equity, Proportionate universalism, Health quality dimensions

## Abstract

**Background:**

Illicit drug overdoses have reached unprecedented levels, exacerbated by the COVID-19 pandemic. Responses are needed that address the increasingly potent and unpredictable drug supply with better reach to a wide population at risk for overdose. Drug checking is a potential response offered mainly within existing harm reduction services, but strategies are needed to increase reach and improve equitable delivery of drug checking services.

**Methods:**

The purpose of this qualitative study was to explore how to extend the reach of drug checking services to a wide population at risk of overdose. We conducted 26 in-depth interviews with potential service users to identify barriers to service use and strategies to increase equitable delivery of drug checking services. Our analysis was informed by theoretical perspectives on equity, and themes were developed relevant to equitable delivery through attention to quality dimensions of service use: accessibility, appropriateness, effectiveness, safety, and respect.

**Results:**

Barriers to equitable service delivery included criminalization and stigma, geographic and access issues, and lack of cultural appropriateness that deter service use for a broad population with diverse needs. Strategies to enhance equitable access include 1ocating services widely throughout communities, integrating drug checking within existing health care services, reframing away from risk messaging, engaging peers from a broad range of backgrounds, and using discrete methods of delivery to help create safer spaces and better reach diverse populations at risk for overdose.

**Conclusions:**

We propose proportionate universalism in drug checking as a guiding framework for the implementation of community drug checking as an equity-oriented harm reduction intervention and as a population health response. Both a universal equity-oriented approach and multiple tailored approaches are required to facilitate drug checking services that maximize reach and appropriateness to respond to diverse needs.

## Introduction

Illicit drug overdoses have reached unprecedented levels in the USA [1, 36] and Canada [40] with illicit fentanyl driving the devastating and ongoing crisis. With the advent of the COVID-19 pandemic, the overdose crisis escalated [20, 23, 37]. The overdose crisis is linked to illicit synthetic opioids, notably fentanyl and its analogues, and has tragically illuminated the limits of current substance use and harm reduction policies and programs. The disproportionate impacts of overdose are well documented with overdoses impacting all sectors of the population [16, 31, 43]. However, equity-oriented responses to overdoses are lacking, particularly equity-oriented services which are contextually tailored to the varied sociopolitical contexts in which people use drugs [49].

Community drug checking is emerging as one innovation necessary within the overdose response given the unpredictable and complex synthetic opioid market [21, 29]. Drug checking provides people with information on the chemical composition of drug samples [32], with a primary aim of engaging people who use drugs in harm reduction [34]. As a response to the current overdose crisis, drug checking has included lower cost and accessible fentanyl test strips, as well as spectrometers including mass spectrometry [47]. However, implementation has been limited [10], and strategies are needed to better reach the wider population that would benefit from these services.

Overdose interventions have been found to be effective; however, overall overdose rates continue to increase [25]. Pardo et al. [38] attribute this to interventions confined to the margins and reaching only a very small proportion of people who use drugs, rather than wide implementation at the population level. For example, supervised consumption sites are a vital and effective innovation but are often limited to inner city sites in large urban areas, and absent in smaller communities [42]. As a foundational step towards equitable service delivery, a public health approach to overdoses is proposed [44] that recognizes and responds to social determinants of health [16]. Moreover, an intersectional risk environment framework is needed that is responsive to the diverse ways in which risks are experienced, both within and across populations, and focusing on interventions and policies for mitigating differential drug-related risks and experiences [13]. Given the overall impact and inequitable distribution of overdoses, it is critically important to ensure scale up of interventions that are widely accessible with broad population reach.

Paired with this need for equitable delivery of overdose responses are calls for innovative responses to address the toxic drug supply as a critical determinant of overdose. Such “supply-side” interventions are crucial in light of the shift to synthetic opioids such as fentanyl, and increasing complexities within unregulated opioid markets [6, 38]. Community-based naloxone distribution, along with opioid substitution programs and prescribed safer supply programs, is seen as vital innovations in overdose responses as well as calls for decriminalization and regulation [4, 7, 14, 26]. While drug checking does not provide an alternative to the unregulated market, it is among the innovations being promoted in recognition of the limits of current safer supply initiatives and decriminalization to intervene in the unregulated market [11, 12, 45]. While drug checking has the potential to improve quality and safety within this context [11, 12, 51], it is currently located primarily within harm reduction services such as supervised consumption sites where reach is limited. Thus, strategies are needed to increase scale and reach to support drug checking as a population level intervention.

We aim to identify barriers to implementation of drug checking services and explore strategies to increase equitable delivery of drug checking services to better reach a wide population at risk for overdose, within the context of an unpredictable and increasingly complex drug supply. In a prior study, we investigated how best to implement community wide drug checking for those accessing harm reduction services [52]. In this paper, we extend our analysis to inform scale up of drug checking services to reach diverse populations that use substances and impacted by the overdose crisis. We sought the perspective of potential service users who do not commonly use harm reduction services, to examine barriers and strategies to increase equitable access to drug checking services and expand the reach of drug checking.

## Methods

We conducted a qualitative study as part of a community-based research project that operates drug checking services within an urban city, Victoria, British Columbia, Canada [48, 51, 52]. The province of British Columbia is considered the epicentre of the overdose crisis in Canada, with overdose fatalities exceeding 43 deaths per 100,000 individuals [2]. These overdose deaths have been linked to potent opioids such as fentanyl and fentanyl analogues, with other adulterants such as benzodiazepines [21, 22, 28].

Our collaborative research team is comprised of university and community partners. The university-based researchers included BW who led the research, primary researchers DH and BP, and a university-based research associate (TvR). The community-based researchers included four members of the local drug user organization, a facilitator from a collaborating harm reduction agency, and a facilitator from the drug checking project. This team has had extensive research experience working on multidisciplinary research projects, and trusted relationships within the community.

The university-based researchers developed the initial research design. We held collaborative sessions with the whole team to review this design and the interview guide and develop recruitment strategies and protocols for consent, data security, and confidentiality. The team pre-tested the interview and relevant prompts. We obtained ethical approval from the Health Research Ethics Board at the Island Health Authority (J2018-069).

### Sampling

In this study, we purposefully sought to understand barriers and strategies needed to implement drug checking for people who may not access existing harm reduction sites and services to inform the development of a drug checking as a population level strategy. While some participants discussed their experience with using reagents and strip tests for drug checking, our interview questions focused on drug checking technologies such as spectrometers. We used multiple recruitment processes such as distributing handbills, posters, and emails, to health and social services that had mandates beyond substance use. We also used third-party recruitment with collaborators sharing recruitment materials with potential participants who then contacted us if interested, or potential participants contacting interviewers within the drug user organization seeking to be included. We provided study participants with a CDN$20 honorarium. While our recruitment strategies were purposeful to reach those who do not use inner city harm reduction services, we used convenience-based sampling and interviewed everyone who expressed interest.

We conducted twenty-six interviews in early 2020. Interviews were conducted by lead researchers BW (*n* = 11) and interviewers from the collaborating harm reduction organization (*n* = 7), and drug user organization (*n* = 8). We collected information on demographics, substance use and overdose. Interviews were recorded averaging 30 min in duration with considerable variation in length (15 min–1.5 h). Graduate research assistants transcribed interviews verbatim, cleaned, entered into NVIVO 11, and wrote analytic memos. The data were stored on a secure shared drive at the University of Victoria.

### Data analysis

We used theoretical perspectives of health equity and dimensions of quality of care to guide our analysis, to better understand what works for whom, in what circumstances. Health equity focuses on unfair and unjust differences in health that are potentially remediable [33, 46, 53]. Equity is concerned with both improving access to appropriate care, recognizing diverse needs and structural determinants of health and ultimately improving health outcomes. In this paper, we focus on the development of more equitable drug checking services to meet the needs of different groups in the population, within the current overdose emergency.

We drew on the BC Health Quality Matrix [3] as an analytical framework that informs equitable access and delivery of quality services. This framework focuses on seven dimensions of quality of care across interconnected areas of health. These dimensions include five related to the individual perspective of the person accessing services: respect, safety, accessibility, appropriateness, and effectiveness. It also includes two dimensions related to systems perspectives: equity and efficiency. We focused on what is needed from the perspective of potential service users. We considered the five dimensions of care for diverse individuals with a wider aim to inform equitable delivery of services from a systems perspective and taking into account wider structural factors. We did not consider the efficiency dimension in this analysis.

We used an iterative process to analyse transcripts. BW and TvR read all transcripts, transcribers’ reflective memos. Our wider research team inductively developed initial themes on a subset of transcripts and assessed them to see how they aligned with and informed on quality dimensions of service delivery. We then examined initial themes using a priori categories based on the BC Health Quality Matrix dimensions of equity, respect, safety, accessibility, appropriateness, and effectiveness. We broadened our conceptualization of the equity dimension to include contextual factors such as structural determinants of health and substance use, and knowledge, attitudes and behaviours of service users that may impact service use. We then coded all transcripts within NVIVO 11 to identify potential barriers and facilitators within each dimension, from the perspective of potential service users. Next, we inductively identified themes within each dimension, informed by our initial analysis. We identified barriers to equitable delivery of services and associated implementation strategies within each of the five individual dimensions, to improve equitable delivery and reach.

## Results

Of the 26 people interviewed, over half identified as a woman, and the majority were aged 30 years or older (Table 1). Around a quarter identified as Indigenous and approximately 40% as lesbian, gay, bisexual, two-spirit, queer or had another identification. The majority were in stable housing, with half having post-secondary training, and 60% reporting incomes above $20,000. While most (22) reported using substances daily and some (4) having overdosed in the last 6 months, only one participant reported accessing a safe consumption site and two a needle distribution program. Therefore, we consider this sample to be primarily a group who do not typically access existing harm reduction services.

**Table 1:** Characteristics of the sample (*N* = 26)

Characteristic	Number (*n*)
*Gender*^a^	
Non-binary/Transgender/Other^a^	1
Man	8
Woman	17
*Age in years*^a^	
20–29	3
30–44	12
45 or older	11
*Identify as indigenous (First nations, Métis, Inuk (Inuit))*	
No	20
Yes	6
*Sexual orientation*^a^	
Lesbian or gay/two-spirit/queer/bisexual/other/do not know	10
Heterosexual or straight	16
*Current level of education*^b^	
High school diploma or equivalence	10
Apprenticeship, trades, certificate or diploma, other certificate, diploma or degree	12
Other	3
*Current living situation*	
Rent house/apartment/townhouse	13
Own house/condo/townhouse	4
Subsidized or supportive housing	4
Other	5
*Personal income*	
Less than $20,000	10
$20,000 to less than $40,000	6
$40,000 to less than $60,000	2
$60,000 or more	6
*Frequency of illicit substance use*	
Daily	11
Weekly	7
Occasionally, not every week	4
Never	4

^a^Some categories were combined to preserve anonymity

^b^There were missing data

**Fig. 1:**
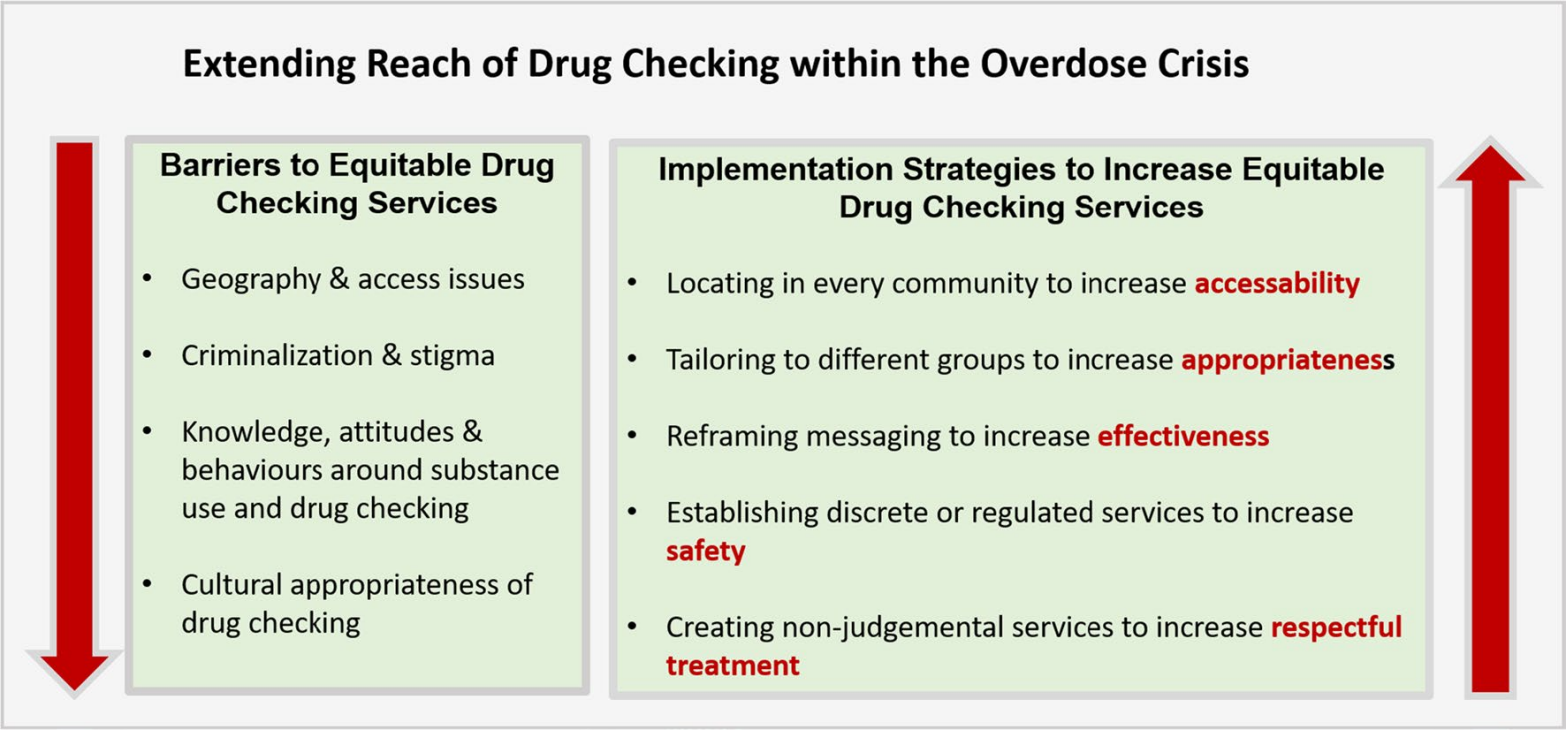
Extending reach of drug checking services within the overdose crisis: barriers and implementation strategies for equitable delivery of community wide drug checking that attend to quality dimensions for services

We present (A): barriers to equitable drug checking services, and (B): implementation strategies to improve equitable drug checking services through attention to quality dimensions of service use (Fig. 1).

A. **Barriers to Equitable Drug Checking Services**

We considered barriers to equitable drug checking services that impede population reach and distribution of services according to population need. Participants reported major barriers to access for themselves and others due to structural determinants including geography, criminalization, and stigma. Barriers also included lack of knowledge and cultural appropriateness of drug checking within certain communities, and individuals’ attitudes and behaviours around substance use and drug checking.

### Geography and access issues

Participants noted access issues in terms of distance from services or lack of services within communities, particularly for rural and remote communities. Poverty was another barrier that created or compounded access issues especially if people have mobility issues, lack adequate transportation, or are required to pay for parking or services. This quote highlighted a number of access issues: “I need free parking … The time of day that it's offered … And I do live far out of town (Q2-18)”. Many participants stated that limited hours would be problematic due to work schedules and other commitments: “Yeah, and the accessibility; you gotta have more than just the three times, 3 days a week, right? (Q2-6)”. Thus, geography and accessibility to services are key barriers to scaling up drug checking services.

### Criminalization and stigma

We heard that critical barriers included fear of repercussions due to criminalization of substances and stigma related to substance use. This included fear of policing and prosecution, as well as fear of being identified as a person who uses substances or a particular type of substance. For example, this participant notes how criminalization deters people from accessing services that might be beneficial:

Due to the fact that people can go to jail for having or selling or possessing substances like that right, but also people don’t want to die, and a lot of people die because they're scared to get caught. So, they don’t access the services. (Q2–19)

Moreover, participants raised stigma and fear of identification as a person who uses drugs as critical barriers. Many participants told us that they did not want friends, family, and co-workers to know about their substance use and therefore they were unlikely to access drug checking services. For example, this participant stated: “I’m sure there’s so many people out there using, that are working, or, and their family doesn’t know. So, I don’t know if they would go to a big drug checking place (Q2-13)”. Participants noted locations associated with substance use would be a barrier due to fear of being identified as a substance user, or to feeling uncomfortable and unsafe as this participant stated: “the venue as well could really put me off. … I don’t know if I would be so comfortable walking into a safe consumption site to get my drugs checked with the any kind of fear of any type of harm I guess (Q2-23)”. Fears of identification may be more pronounced in a small community where privacy accessing services are more limited: “Well basically a lack of privacy. Like, if it was in a tiny community and everybody knows. … yeah just a lack of confidentiality and privacy in a small community (Q2-4)”*.* Fear of criminalization and stigma deterred participants from using drug checking services.

### Cultural appropriateness

We heard how drug checking services may not be culturally appropriate which render them less accessible and welcoming for certain groups in the population. For example, participants indicated that drug checking may not be welcome in some Indigenous communities: “I think for the reserve, its going to be really tough because their initial agenda is if you use drugs if you sell drugs here, you’re kicked off the reserve. It’s not as acceptable yet to be a drug user (Q2-5)”*.* Thus, drug checking may not fit for the community, and regulations may deter use.

### Knowledge, attitudes, and behaviours around substance use and drug checking

Participants cited lack of awareness about drug checking and its potential benefits as a barrier: “just the awareness of people knowing that it’s out there and that it’s an option (QR-33)”*.* Participants also noted how behaviour around substance use could be a barrier. For example, if people want to use quickly after purchasing a substance, testing may be less likely due to the time involved. The participant below describes how their purchasing patterns influence access to drug checking services.

My mindset is festival drugs. Sort of like psychedelics, um, edging on like MDMA or cocaine. I personally tend to buy drugs sort of in larger quantities so that I reduce the number of times that I am exposing myself to risk. So, I’d very rarely be like buying drugs on the street, testing them, and then doing them in very short order (Q2-8).

Similarly, several participants noted possible differences in likelihood of testing based on type of substance use or other personal circumstances as the above quote highlights.

B. **Implementation strategies to increase equitable drug checking services**

We identified implementation strategies to address these barriers and increase the equitable delivery of drug checking services through attending to five quality dimensions of service delivery: accessibility, appropriateness, effectiveness, safety, and respect. Overarching themes within each dimension included: “Locating in every community to increase accessibility”, “Tailoring to different groups to increase appropriateness”, “Reframing messaging to increase effectiveness”, “Establishing discrete or regulated services to increase safety”, and “Creating non-judgmental services to increase respectful treatment”.

### Locating in every community to increase accessibility

To create ease of access, different groups may require different strategies, with commonalities being that services are fast, free, and available all hours. Our findings suggest locating services where people live or use substances, reaching people at home, and providing non-contact options can increase ease of access for different groups and communities.

Participants noted that centralized services are important for some, in particular those who are street involved and/or living downtown and potentially already accessing available harm reduction services. However, we heard this didn’t fit for others such as those living in suburban or rural areas. One participant indicated that drug checking services need different strategies to reach different populations: “I think it kind of needs two branches. Like of the operation. You need the people to stay in the safe consumption site because that's for sure they need that. And then you need to have outreach for the suburban people. I don't have a name for it yet, but basically like the rest of the community [Q2-19]”*.*

Participants highlighted that for many, centralized services were not considered convenient enough to be a viable option:

if you’re hoping to capture people who are in a more suburban based setting I imagine some of the challenges are like you know if drug checking is only offered downtown it’s if somebody is driving out to like [municipality] or [city] you know it’s tricky, you know and these people are like working their like 9 to 5, when is kind of the ideal time I imagine that you can get them. I think we can all be pretty basic of like is this close enough to me, is it convenient for me, does this like fit my schedule, you know those things can show up as little barriers, I don’t know if they’re deal breakers but they definitely impact they show up [QR-21].

To increase reach, participants noted that drug checking services need to be widely available where people live and work. They suggested locations that are common in most communities and widely used such as walk-in or STI clinics, medical laboratory test sites, or pharmacies: “You don’t need a car to get to a walk-in clinic, you don’t need to go downtown. You can stay in your local neighborhood, kind of a chicken and the egg problem, you kind of need them everywhere for them to become like usable (Q2-8)”.

In addition, we heard that to reach people at home strategies such as mobile services, take home testing, and runners who take drugs to be tested could reach populations not currently accessing downtown harm reduction services. For example, one participant suggested readily available at home testing for those who consider accessing services inconvenient:

They don’t want to get up and go and, you know, it’s going to take an hour at least to get this task done. They just want their buddy to come over, test it, and get out of there. And that – you know, like make it a fifteen-minute process rather than an hour to ninety-minute process or longer. And not cost them anything. Like they don’t have to expend any energy they don’t need to get a bus ticket or gas or whatever [Q2-15].

Services such as mobile drug checking may also help to reach those in the suburbs or those with access issues:

I think maybe a drug pick up van, somebody that goes from house to house, picks them up and labels them as sample A, B, or C. And then drops the samples results off, would be a valuable service such as the needle replacement service that was a drug van. That could be a definite option because there are some people that are lower income that have issues and troubles getting to certain locations [X1-1].

Such strategies may also help people who have mobility issues that make distance or accessing certain locations challenging.

Participants also suggested non-contact options like auto-test machines, drop boxes, mail in services, and email/text/online results as access strategies to increase convenience: “It would be 24 h a day. It would be just like an ATM machine, no people, you just take a little bit put in the machine and it spits out the results [Q2-18]”.

This highlights the critical importance of strategies that focus on convenient easily accessible services, with drug checking available in every community through a variety of methods of delivery. This universal approach to drug checking services, in addition to targeted and centralized approaches, is required to better extend reach and enhance equitable access to services.

### Establishing discrete or regulated services to increase safety

We considered how to ensure that drug checking services were safe spaces. We identified fear of identification by family, friends, employers or law enforcement, or persecution as a primary barrier to safety. Participants recommended strategies to ensure services were anonymous and discreet, as this participant advocated: “make the drug testing as discrete as the drug deal until its legal Q2-22”. Alternately, we heard that services that appeared as government regulated could help to increase feelings of safety.

Participants suggested mobile outreach, the use of third-party checkers or “runners”, online results with encrypted messaging and other non-contact methods as discrete ways to reach people who do not want to be identified as using drug checking services or do not feel comfortable within certain spaces. A participant made the analogy, “you know like a typical housewife who doesn’t want to go to certain areas of town. …Like having something like a mobile service would be useful (Q2-3)”*.* Moreover, participants noted that they didn’t want to be asked why they were using services, for whom, or about their substance use: “I think they shouldn’t be asked. Is it your drugs, is it someone else’s? I mean you are there for testing the drugs. You know, I don’t think they should need to even go there as to where the drug came from (Q2-13)”*.*

We also heard that seemingly public places such as drug stores, walk-in medical clinics or community laboratories could be considered preferable because they are not associated with substance use and used by everyone for many purposes: “Something that isn’t dedicated for that specific use, like you’re walking in that building and they knew you were going there (Q2-7)”. This offers protection from identification if services can be offered discretely in these settings. For example, when discussing the need for discrete services, this participant stated: “that sort of experience where it is totally anonymous. You walk in, you could be walking in for any number of reasons. You’re taken to a room and it’s all private (Q2-8)”.

Participants mentioned professional storefront approaches could also increase the appearance of legitimacy and make people feel safer to use them without possible criminal repercussions:

One of the most interesting aspects of cannabis legalization in BC is how like professional the storefronts are. You know, they are computerized, like, it feels very official and allowed. Stark difference than you know meeting someone on a street corner and like exchanging something. And that image and aesthetic and like semiotic of just like a professional service, with corporate investment, provides a lot of reassurance to me, that this is OK, that I should use this service, that I can use this service, that I won’t suffer any like consequences for walking through these doors. (Q2-8).

Hence to extend the reach of drug checking services, it is critical to attend to fears of identification created by criminalization and stigma. Strategies include the use of common venues and low-contact methods of delivery that are not associated with substance use to provide anonymity that creates safety for service users, or services promoted as government regulated. Recognition is needed that different spaces will feel safe depending on an individual’s circumstances.

### Tailoring to different groups to increase appropriateness

We heard that drug checking needs to be “accessible for different people in different ways [Q2-19]”, with strategies such as integrating into existing services that people already rely on for care, and considering where people use substances when planning how best to reach different groups.

Participants indicated that integrating drug checking with a wide range of harm reduction and health care services was a useful strategy to increase reach. However, these services may vary depending on what substances people use, where they are comfortable accessing services, and from whom they are comfortable accessing services. For those already accessing harm reduction services, participants noted these are necessary locations that work well but that services need to be expanded to better reach others who may not access harm reduction services. For example, “the dealers who want to remain anonymous, the working class that are doing it in secret. You know moms who don't want to lose their kids the other people basically (Q2-19)”.

For those not accessing harm reduction services, many expressed discomfort with using such services:

I feel like it depends on the spectrum of the person who’s walking in there, if you have somebody who’s like “oh I just want to like grab a like grab a bag of coke and have a party with my friends on the weekend” vs somebody who’s like a chronic fentanyl or heroin user who you know is maybe accessing different shelter spaces there can be like, there’s a different, there can be a different experience of youth and of access and so you know the person who is going to this party on the weekend could be like, “oh this isn’t really maybe a space that I should come into” or maybe there might be a little bit of hesitation or fear or uncertainty or all those things that come up when it’s already like hard enough (Q2-21).

To address this, we heard that integrating drug checking within health-related services that people already use and feel comfortable with would work well: “if it was like embedded in our healthcare system somehow (Q2-19)”. Suggested locations included medical clinics, public health units, laboratory test services, and pharmacies. For youth, participants suggested schools, counselling services, and youth clinics. Further, linking drug checking to current medical care would facilitate use of the service through existing trust: “if [my doctor] said to me ‘ok well I I've heard about this drug checking service that’s available would you like some information on that’? That coming from my doctor would speak volumes, like you support this drug checking, well ok you’re the one who manages my health and I appreciate you mention it and yeah I would be much more inclined to check it out (Q2-23)”.

Participants noted that that to better reach those not accessing services, it would be helpful to provide services where people use substances. This will vary for different groups and require different strategies. Participants recognized the importance of providing services at shelters or in parks through mobile outreach, as well as locating services where recreational substance use may be common such as festivals, parties, events, bars, or washrooms.

Thus, a critical strategy to develop appropriate drug checking services includes integrating into harm reduction and health care services that people already feel comfortable accessing. Given intersecting identities and substance use across every demographic group, consideration is required to provide comprehensive and cohesive services tailored to diverse needs. Furthermore, locating services where people use may also better tailor services to meet needs.

### Reframing messaging to increase effectiveness

We considered how to develop drug checking services that benefit service users by providing information on the composition of their substance to inform personal harm reduction. Participants noted that a focus on the harms and risks of substances is not effective and can increase hesitation to test by reinforcing existing stigma. We identified this an issue across diverse populations, including for those in the suburbs who may be hiding their use and prefer to use alone, and those who use recreationally and do not perceive risks to be high. We identified that reframing drug checking messaging away from risks and harms to greater drug information and knowledge would be an important strategy for promoting the potential benefits of drug checking to better resonate with different groups and circumstances.

Participants suggested shifting messaging to establish drug checking as part of a culture that simply provides information, or potentially taking a more “*playful*” approach would be strategies to counter stigma and extend the reach of drug checking:

Perhaps a little bit more playful, perhaps a little bit more engaging … like a lot of sexual health testing does or like STI checking and especially when it’s done in queer communities in times when there's like been acts and different things that have been like really, things have been really sexually repressed or like stigmatized or like we’re going to get a little bit more cheeky with our language or going to be a little bit more bold and playful here to be like hey we’re laughing and you can laugh with us. (Q2-21).

For others, such as those within the supply chain, we heard that a focus on quality may be more effective, whereas we heard that framing drug checking as caretaking would be a way to reach family or friends who may wish to help others who are not likely to access services themselves. One participant highlighted that people need to see themselves in promotion for drug checking and suggested “a poster campaign or something like that of who like different kinds of profiles of people that really like represents people that could really benefit from drug checking away from like high school student, from dealer, to somebody who’s going to festivals, to somebody who’s living in shelter spots to parks, to really create a bit of a different representation of it (Q2-21)”.

Overall, this highlights the diverse groups that can benefit from drug checking services, and that risk reduction messaging and a focus on harms may not be an effective strategy if it reinforces stigma or does not appear to offer value and deters people from accessing services. To be effective, messaging needs to align with people’s perceptions and preferences around substance use and how drug checking can provide benefits in their circumstances.

### Creating non-judgemental services to increase respectful treatment

We overwhelmingly heard the critical need for services to be respectful and free from judgement to reduce stigma and discrimination associated with substance use.

Participants emphasized the importance of staff who operationalize fundamental principles around harm reduction and support service users with acceptance and respect. Participants suggested people with lived experience of substance use as suitable staff to increase trust and respectful treatment.

We heard that staff need training in cultural safety and harm reduction and to accept that abstinence is not the aim of drug checking services. One participant stated, staff should be “taught that that you can’t discriminate against people that are coming in (Q2-2)” and that judgmental treatment will increase risks not deter substance use. “I think that, just the dedication to no judgement is key, because, I don’t think judging somebody on it is going to stop them from using. I think that it’s just going to stop them from getting checked if it’s safe to use it (Q2-7)”.

Participants identified people with lived experience as potential staff who would facilitate trust and who participants felt would treat them with respect: “I think that there's probably that need for a maybe like a substance user, or somebody who’s very empathetic to be able to have that that interface with the people who are dropping off the drugs. Somebody who's not going to pass any judgement or look at you sideways if you bring in something really bad or really kind of tweaked out when you go in there. Somebody who’s very receptive to the community, I guess (Q2-23)”.

However, we heard that there are many different people who can fit the definition and role of “peer”, and that the capacity to provide non-judgmental, respectful services was not limited to peers:

I don’t think it's an absolute prerequisite … to be a user… and if not then at least have a really strong passion or engagement or excitement about learning about just different substances the way they work. And also understanding that this is like the way it exists. Maybe somebody kind of situated in like an anti-oppressive lens and in a way that that takes into account the many different reasons why people are choosing to take drugs and for someone who can really just like offer that open space (QR-21).

Thus, it is critical that staff receive adequate training in cultural safety and harm reduction and building capacity for respectful, non-judgmental care is essential. Furthermore, staff with lived experience from a range of backgrounds can help to create trusted and respectful services.

## Discussion

We sought the perspectives of potential service users from a range of backgrounds to explore strategies to enhance equitable delivery of drug checking services to a broad population at risk of overdose. Critical barriers to equitable service delivery included criminalization and stigma resulting in fear of identification and other repercussions, geography and access issues, and lack of cultural appropriateness. When seeking to explore strategies to address these structural issues, participants reported extremely diverse needs, preferences, and personal barriers for themselves and others. We heard that centralized services within harm reduction services are important. However, drug checking needs to be located widely throughout communities to increase access and convenience. We identified potential strategies to better reach those who may not feel comfortable accessing existing services or see benefits for their circumstances. These included integrating within existing health care services; reframing messages away from risk messaging to messaging around substances; engagement of peers from a broad range of backgrounds in services; and use of discrete methods of delivery such as non-contact, mobile or locating in common sites within communities not associated with substance use. This highlights the need for universal access through multiple tailored approaches to maximize reach and appropriateness for a wide population at risk for overdose.

A potential strategy is integrating drug checking into existing services where people already feel comfortable accessing care and trust has been established. Participants recognized and affirmed the importance for drug checking services being co-located with other harm reduction services such as supervised consumption sites as has been noted previously [10, 50]. However, we illustrate that sole reliance on targeting of drug checking to perceived high-risk or high-need populations can reinforce the stigmatization of substances and people who use substances and is not necessarily safe or accessible spaces for all people who use drugs. When drug checking is embedded in inner city health and social services, the stigma of substance use as an inner city problem may be reinforced. Further, this may exclude those outside of the inner city who do not use shelters and other harm reduction services or have access issues related to mobility or transportation. To create services appropriate to better reach at a population level, we determined that integrating drug checking into other health care services which people already rely on for care can be a strategy to increase equitable delivery of services. This would also ensure a more comprehensive and cohesive model of care model for those with complex needs.

Participants also highlighted the need to create safer spaces that offer protection from identification as a drug user and other repercussions. They suggested the use of commonly accessed sites such as pharmacies as well as non-contact options, and mobile services to help increase discretion and anonymity. The use of common sites not associated with substance use within communities, and pairing with existing health care services, potentially offers feasible ways to increase scale and reach.

We also overwhelmingly heard the need for respectful services that are culturally safe and grounded in principles of harm reduction. Care must be taken to ensure that training and capacity for delivering safe and respectful care exist within all models of delivery. These strategies offer ways to mitigate risks within the current context of criminalization and high levels of stigma related to substance use. Broader structural policy interventions such as decriminalization are needed to address this context and would reduce barriers to drug checking.

Drug checking’s mandate and messaging should also reflect universal principles of a right to know and quality control, rather than solely detecting and reporting risks and harms [9, 51]. An exclusive focus on the risks of substances and mitigating harms in drug checking messaging feeds into stigmatization and is less accepting of substance use that is not as inherently harmful. Similar to other arenas for prevention, there is a role for messaging with a focus on pragmatics and pleasure within substance use [17, 18, 35, 41] or with more “playfulness” (as noted by one participant) to help break down stigma and better resonate with service users. This is useful to consider for both promotion of services, and in service delivery and messaging of results. Locating services where people use, such as festivals or events, and aligning with the culture fits with this recommendation. The long history of drug checking within festival environments aligns with this approach and offers another example of how drug checking can be positioned for greater acceptance and reach within different communities.

Our findings reflect the need for drug checking to function at a meaningful scale by reaching a broad population of people who use substances and who may benefit from services. Participants defined scale and reach as widespread geographic accessibility, culturally appropriate services, and mitigating the contextual barriers of criminalization and stigmatization of substances and people who use substances. Overall, there was a general recommendation to ensure universal access while tailoring of services to best respond to the unique needs and values of differing groups, and the need for engagement when defining and delivering services. This is of particular relevance when considering services within Indigenous communities, where there may be policies that prohibit substance use as participants noted. Given the complex historical relationships between criminalization, colonialism, and substance use perpetrated against Indigenous peoples and communities, engagement with Indigenous communities and elders is a critical first step in exploring whether such services are appropriate, and if so, how best to deliver them, while taking into account unmet need from people who identify as Indigenous [15, 30, 31].

We perceive overdose interventions, including community drug checking, to be primarily targeted interventions to those perceived to be most at risk. This is often inner city, homeless, or precariously housed adults who overall fit criteria of experiencing structural violence and system inequities and assumptions of being marginalized [26]. Meanwhile, the everyday use of illicit substances is much more pervasive, with coroner inquiries confirming that lives are being lost across the social gradient and geographical regions. Indeed, in the jurisdiction in which this research took place where overdose fatality rates exceed 40 per 100,000, public health acknowledges [5] they are not reaching all those at risk. This includes those who are fatally overdosing in their homes; notably, men, youth and young adults, and often concealing their substance use from spouses and family in the residence in which they die.

Based on this unmet need and our findings from this study, we argue for proportionate universalism [8, 19] as a guiding framework for the implementation of community drug checking as an equity-oriented harm reduction intervention. Proportionate universalism suggests that interventions “must be universal not targeted, but with a scale and intensity that is proportionate to the level of disadvantage” (pg1) [8]. This is termed by others as targeting within universalism [19]. However, practical definitions of proportionate universalism vary, and some may not bring an equity lens to the application of policy and services [19]. We propose this framework for drug checking to provide universal access to the general public where substance use is endemic, delivered through multiple tailored approaches to maximize reach and appropriateness for addressing structural inequities and unique needs and values. This approach must be grounded in recognition of structural determinants of substance use and harms such as criminalization, colonialism, and stigma, and how social positions and systems intersect to impact both service use and health [39, 46].

While such universal approaches were recommended, people also advocated for targeted sites, services, and approaches that cater to unique needs and provide more appropriate services for diverse groups. Such approaches reflect the principle of particularism within universalism [8], in which the unique identities of groups are central and worthy of particular approaches as opposed to deficits requiring targeting. To create such balance in a framework for proportionate universalism in drug checking, people raised examples of the principle of subsidiarity [8] which resembles harm reduction’s principles [24] of meeting people where they are at, providing pragmatic responses, and “nothing about us without us” [27]. As a governance principal for drug checking, we recommend culturally safe and trauma-informed services provided by peers. We highlight the need for approaches that reflect the universal population of people use substances, while ensuring tailored services engage peers from across social groups.

Overall, our findings reflect proportionate universalism’s emphasis on upstream vs downstream priorities [19]. Drug checking can reflect upstream principles by recognizing substance use as a universal part of society and life, creating evidence to inform policy change, and problematizing drug laws that reduce the security of substance use. Upstream approaches support universal access to drug checking for all people who use substances. This requires the wider distribution of services in common locations where the general public access everyday needs, as well as selective approaches tailored to reach unique groups. Lastly, an upstream approach tailors services to cater to people who produce and sell substances for interventions to have upstream impacts in the market.

Proportionate universalism recognizes and responds to the social gradient in health [8, 19]. Within drug checking, our findings reinforce the need to respond to the gradient of needs through targeting and tailoring, while providing universal access, to reach a broader population regardless of social status, income, and geographic location. We do not see proportionate universalism in drug checking as focused on reducing the gradient, but rather reaching and responding to the gradient in substance use. We recognize there are unique barriers along the gradient, and drug checking can balance such priorities while operating at a meaningful scale.

## Strengths and limitations

A strength of this study is the focus on perspectives of people who are eligible to use drug checking services as to what is needed to implement drug checking to extend reach across the population. This included those who use and sell substances or are interested in testing for others. Our research team included community partners and academic researchers with established relationships in the community which facilitated trust and recruitment. The study was based in Victoria, BC, where drug checking is currently operating as part of the overdose response and the burden of overdose is high. We used theoretical perspectives of health equity, and a framework that prioritizes service user perspectives, to guide analysis and ensure consideration of broader contextual factors that can be critical barriers to implementation. Limitations include challenges in recruiting men in the trades, who have been disproportionately affected by the overdose crisis and may not be accessing harm reduction services, and youth. Furthermore, some participants had limited knowledge of drug checking. Some of the community researchers with the local drug user organization were no longer associated with these organizations, or their roles changed during COVID-19, which affected their engagement post data collection. While we provided interview training and supports, there was variation in interviewing abilities and some interviews were lacking depth. Future engagement with communities is needed to consider whether drug checking can be implemented in culturally appropriate ways within Indigenous communities, and how best to do this to balance needs of individuals and communities.

## Conclusion

Overdose responses need to be implemented at the population level to address the overdose crisis and the broad population at risk. Drug checking has the potential to improve quality and safety within a potent and unpredictable drug market; however, implementation has frequently been limited to existing harm reduction services and sites. To extend the reach of drug checking, a universal equity-oriented approach is required that can attend to unmet needs and enhance overdose responses. This must be grounded in recognition of structural determinants of substance use and harms such as criminalization, colonialism, and stigma, as well as geography and other access issues that create diverse circumstances and needs. While critical, sole reliance on targeting of drug checking to perceived high-risk or high-need populations can reinforce the stigmatization of substances and people who use substances and does not necessarily create safe spaces. Universal access, through multiple tailored approaches that are culturally appropriate and safe, is needed to improve equitable delivery of services for the wider population at risk of overdose as well as those with the most structural disadvantage. We propose proportionate universalism in drug checking as a framework to provide universal access to the general public where substance use is endemic, delivered through multiple tailored approaches to maximize reach and appropriateness for addressing structural inequities and unique needs and values.
